# AI-based fluid quantification in neovascular age-related macular degeneration and diabetic macular edema treated with faricimab – a real-life study

**DOI:** 10.1007/s00417-025-06834-5

**Published:** 2025-09-26

**Authors:** Anna Theresa Lorenz, André Marcio Messias, Warda Darwisch, Philipp Ken Roberts, Karl Thomas Boden, Peter Szurman, Boris Viktor Stanzel

**Affiliations:** 1 Eye Clinic Sulzbach, Knappschaftsklinikum Saar GmbH Krankenhaus Sulzbach, An d. Klinik 10, 66280 Sulzbach, Saar Germany; 2 Klaus Heimann Eye Research Institute, Sulzbach, Germany

**Keywords:** Faricimab, NAMD, DME, Fluid quantification, Artificial intelligence (AI), Optical coherence tomography

## Abstract

**Purpose:**

To assess faricimab treatment in neovascular age-related macular degeneration (nAMD) and diabetic macular edema (DME) with artificial intelligence-based fluid quantification on spectral-domain optical coherence tomography (SD-OCT).

**Methods:**

Retrospective patients with nAMD and DME; treatment-naïve, or on another intravitreal medication (switchers), loaded with 4 monthly intravitreal faricimab. SD-OCT (Heidelberg Engineering) at baseline and 16 weeks, then processed using the Fluid Monitor® (RetInSight). Sum of fluid volumes in the central 1 mm (pigment epithelial detachment, subretinal fluid, and intraretinal fluid) was computed (SF) and correlated with central subfield thickness (CST).

**Results:**

Thirty-four nAMD (25 switchers) and 21 DME (20 switchers) eyes were included. SF (nL) was 126.68 ± 17.24 and 37.84 ± 8.31 at baseline reduced to 80.78 ± 15.56 (*p* < 0.0001) and 15.28 ± 4.94 (*p* < 0.0001) for nAMD and DME, respectively. CST (µm) reduced from 405.12 ± 24.95 and 354.97 ± 15.89 to 320.33 ± 19.80 (*p* = 0.0001) and 302.41 ± 11.55 (*p *< 0.0001) in nAMD and DME, respectively. Mean intraindividual change between baseline and 16 weeks was larger using SF than with CST for nAMD (36.5% and 17.6%, respectively) and for DME (56.2% and 13.1%, respectively). A similar pattern was observed for each retinal compartment.

**Conclusion:**

When loaded with faricimab, total fluid decreased by 37% in nAMD and 56% in DME. Fluid volumetry appears more sensitive for retinal fluid.

**Translational relevance:**

When AI-fluid volumetry is applied real-world nAMD and DME cases, then fluid volume offers a more sensitive and quantifiable measure of disease activity than CST.

## Key messages

***What is known***

• Retinal fluid exudation is a key biomarker for disease activity in neovascular age-related macular degeneration (nAMD) and diabetic macular edema (DME). Central subfield thickness (CST) is widely used as a proxy for the latter but has limitations in sensitivity and reproducibility.

• Faricimab has demonstrated efficacy in reducing retinal fluid and CST, showing potential superiority in fluid resolution compared to existing treatments..

***What is new***

• This study showed AI-based fluid volumetry (Fluid Monitor®) as a more sensitive and quantifiable measure of retinal fluid compared to CST. Fluid volume reductions were notably 2 and 4 times larger than CST reductions for nAMD and DME respectively, thus offering improved sensitivity for assessing treatment efficacy.

• The presented real-world data revealed a significant fluid reduction after faricimab upload, with a 36.5% reduction in nAMD and 56.2% in DME.

• Treatment-naïve nAMD patients showed greater reductions in total fluid volume (49%) compared to switch patients (32%), suggesting a more pronounced anatomical response in previously untreated eyes.

## Introduction

Neovascular age-related macular degeneration (nAMD) and diabetic macular edema (DME) represent two major causes of vision impairment and legal blindness in the elderly and diabetic populations worldwide. The pathophysiology of both diseases involves the breakdown of the blood-retinal barrier, leading to the accumulation of fluid within or under the retina, which if not managed promptly and effectively, can lead to irreversible damage to the macula causing permanent vision loss [1–3].

In recent years, the management of these retinal diseases has been revolutionized by the advent of anti-vascular endothelial growth factor (anti-VEGF) therapies [4, 5]. Faricimab, a novel bispecific antibody, has emerged as a significant advancement in this domain. Unlike traditional anti-VEGF treatments, faricimab targets angiopoietin- 2 (Ang- 2) in addition to VEGF-A, which are both critical in vascular instability, inflammation, and leakage that contribute to the disease pathology of nAMD and DME. Clinical trials have underscored the efficacy of faricimab, not only by demonstrating non-inferiority to existing treatments for visual acuity, but particularly by showing superior fluid resolution, thus extending the durability of treatment effects, thereby reducing the treatment burden on patients with nAMD and DME [6, 7].

The quantification of retinal fluid, a key indicator of disease activity, is paramount in the management of nAMD and DME [8, 9]. Traditionally, fluid assessment has been predominantly reliant on optical coherence tomography (OCT), interpreted binary- by presence only- a subject to bias by clinicians. This can be time-consuming and subject to inter-observer variability, which was found to cause a significant number of undertreatments. In recent years, the integration of artificial intelligence (AI) into ophthalmic care has begun to revolutionize the diagnosis and management of retinal diseases [10, 11]. AI algorithms, particularly those based on deep learning, have shown significant potential in accurately quantifying intra- and subretinal fluid from OCT scans. This capability is crucial, as the amount of fluid in the macula serves as a vital biomarker for disease activity and treatment efficacy [12–14].

Artificial intelligence systems are trained on large datasets of expert human annotated images to detect and quantify fluid with a high degree of precision [15]. These systems can support clinical decision-making by providing consistent assessments, which are crucial for tailoring treatment plans, especially when using newly established therapies like faricimab. Moreover, AI-driven tools can predict treatment response by analyzing subtle changes in fluid levels and retinal morphology over time, thus potentially preventing overtreatment or undertreatment [12, 16]. The real-life application of AI in fluid quantification for patients treated with faricimab presents a novel area of research. This study aims to enhance our understanding of fluid dynamics in real-world settings by using an AI algorithm (Fluid Monitor®, RetInSight, Austria) to automatically quantify macular fluid on SD-OCT in patients with nAMD or DME first treated and/or switched to faricimab.

## Methods

### Study overview

This was an observational retrospective study conducted at a single center (Eye Clinic Sulzbach, Germany). The study involved a retrospective analysis of patients’ data, collected as a part of quality control from clinical routine for patients with nAMD or DME since October 2022 treated with intravitreal faricimab (Vabysmo®, Roche/Genentech) injection. The study was carried out in accordance with the tenets of the Declaration of Helsinki, including current revisions, and written informed consent was obtained from all patients. The research plan and relevant supporting information was reviewed and approved by the IRB/EC (Ethikkommission Ärztekammer des Saarlandes, Votum 221/23).

### Patient selection

Eligibility criteria included adults diagnosed with active neovascular age-related macular degeneration (nAMD) or diabetic macular edema (DME) who received a regimen of four monthly intravitreal injections of faricimab. Participants within each group were categorized as:

• Treatment-naïve: Patients who initiated treatment with faricimab without any prior intravitreal injections or have had no injections for more than 6 months.
• Switch patients/switchers: Patients who received anti-VEGF or steroid intravitreal injections within six months prior to their first faricimab injection.

Treatment regimen involved four monthly intravitreal injections of faricimab at baseline and at weeks 4, 8, and 12. During each visit and prior to the injection, patients underwent a standardized ophthalmologic examination including corrected distance visual acuity (CDVA), slit lamp biomicroscopy with fundoscopy and intraocular pressure (IOP) measurement.

SD-OCT imaging had to be available for baseline and the 16 week follow up visit. Patients with ungradable OCT scans and patients with other coexisting macular diseases were excluded.

### Imaging protocol and data collection

**Fig. 1:**
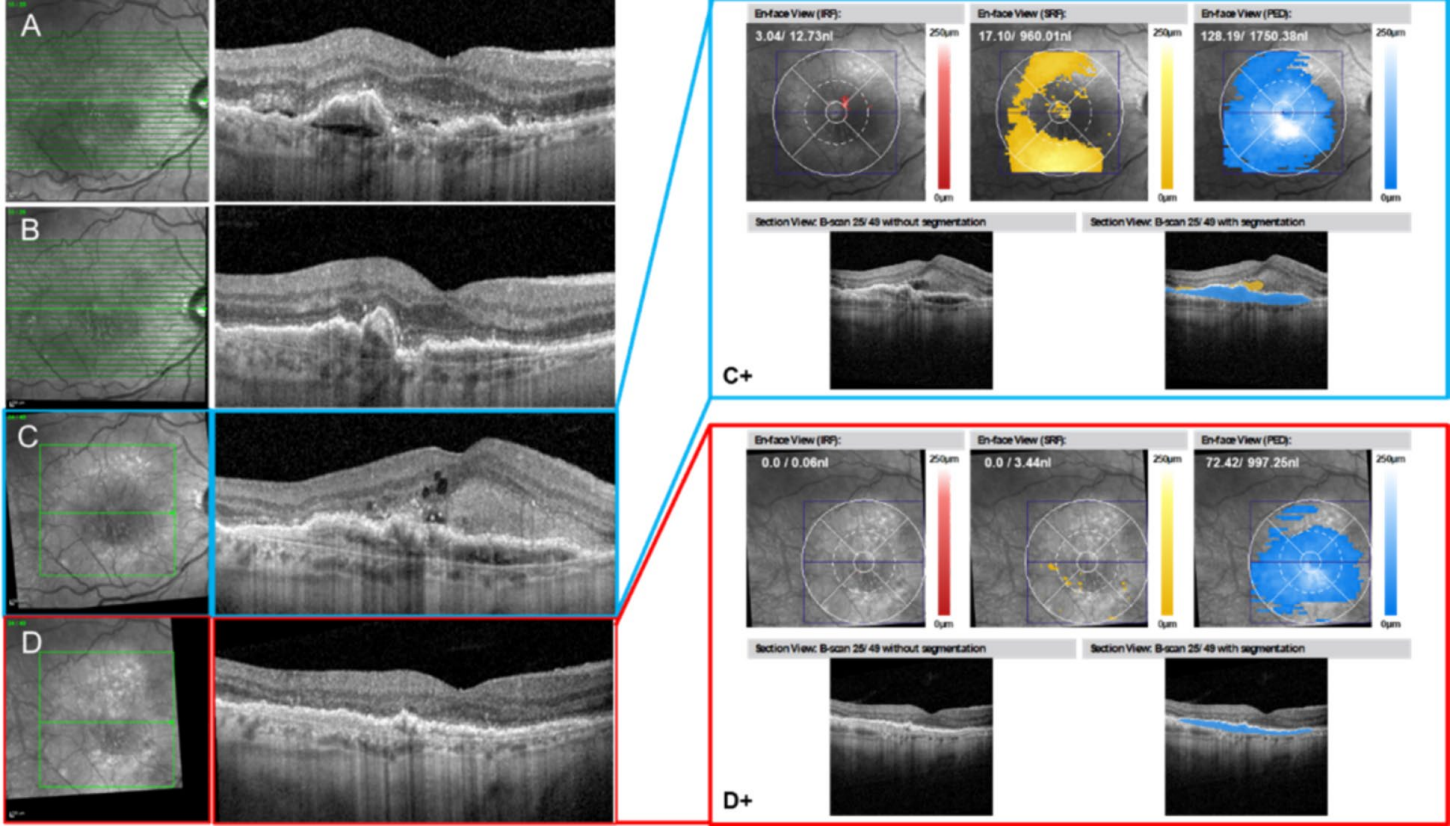
Example case for nAMD (male, 75y/o). **A** baseline showing PED, SHRM/SRF. **B** Following upload with 3 monthly ranibizumab at 12 weeks from baseline, there’s substantially reduced SRF, however a seemingly enlarged PED. The patient was then lost to follow up for 15 months. **C** Baseline prior to faricimab showing fluid in all 3 compartments, which are quantified with the RetInSight Fluid Monitor in (C +). Following upload with 4 monthly faricimab, there’s significantly reduced fluid in all 3 compartments, which is quantified in (D +). These fluid monitor measurements were used in for an nAMD (on label) and DME cohort (off label)

Spectralis SD-OCT (Heidelberg Engineering, Heidelberg, Germany) scans were performed. The acquired images were 6 × 6 mm, 20 × 20 degree, with a density of 49 scans (120 µm apart), averaged 15 times (ART mode set to 15). Measurements of central subfield thickness (CST) were taken within the central 1-mm of the ETDRS grid centered on the foveal center. The Spectralis software as well as the automated readout from the fluid monitor display CST as the vertical distance between the internal limiting membrane (ILM) and Bruch’s membrane (BM). These scans were captured initially at baseline and subsequently at the 16-week visit to assess changes over the treatment period. AI software (Fluid Monitor®, RetInSight, Vienna, Austria) was utilized to post-process these images, namely for automated read out of CST, IRF, SRF and PED (Fig. 1).

The Fluid Monitor® algorithm had received approval as a medical device (class IIa/MDR 2017/745/EU) specifically for patients with nAMD. However, the original deep learning neural network algorithm of the Fluid Monitor® has undergone repeated training on DME datasets, thus enabling its off-label utilization in the current research study.

### Quantification of fluid volume

The AI-based Fluid Monitor® algorithm categorizes and segregates specific pixels within the central 1- and 6-mm macular area (EDTRS). Fluid volumes within this region can be quantified and stratified into three distinct (disease) compartments:

• Intraretinal fluid (IRF): Accumulation of fluid within the layers of the retina.
• Subretinal fluid (SRF): Fluid accumulation between the retinal pigment epithelium (RPE) and the neurosensory retina.
• Pigment epithelial detachment (PED): Accumulation of fluid beneath the RPE.
• Sum of all fluid compartments (SF): Refers to IRF, SRF, and PED for nAMD group, and IRF and SRF for DME group.

The software calculates the number of pixels corresponding to each type of fluid, allowing for an estimation of fluid volumes in nanoliters and their respective locations (Fig. 1). The duration of this process varies depending on the volume of OCT data being processed and server structure present, typically taking between 1 to 5 min.

### Outcome measures

Primary outcomes included:

1. The overall drying effect, defined as total change in sum of fluid (SF) in all compartments within the central 1 mm, versus the change in central subfield thickness (CST) at 16 weeks, following an upload of four monthly faricimab injections.
2. Drying effect stratified by specific fluid compartments (IRF, SRF, PED) at 16 weeks.

Secondary outcome measure was change in corrected distance visual acuity (CDVA), which was extracted from electronic medical records.

All variables were analyzed for both nAMD and DME groups. Additionally, within the nAMD group, a further sub-analysis comparing treatment-naïve and switch patients was conducted.

### Statistical analysis

Descriptive and inferential statistics were performed to evaluate patient characteristics and the changes in CST, SF, and IRF, SRF, and PED volumes, alongside CDVA. Standard linear regression analysis was used to explore the relationship between SF and CST at baseline and 16-week visits.

The overall comparison of changes between timepoints were analyzed with Wilcoxon Signed Rank test and a p-value of < 0.05 was considered statistically significant. Due to the multitude of hypothesis tests conducted, the Bonferroni correction was used to safeguard against erroneous conclusions stemming from false positive results. All statistical analyses were conducted on SAS software.

## Results

In this retrospective real-life study, we analyzed the impact of 16-week intravitreal faricimab injections on retinal fluid levels in 55 eyes using AI-based fluid quantification software.

The study included 34 eyes with nAMD (25 switchers) and 21 eyes with DME (20 switchers). A total of 47 patients were enrolled (29 males and 18 females; mean age 71.9 years ± 11.03); 31 patients (19 males and 12 females) had nAMD, and 16 patients (10 males and 6 females) had DME.

Prior to switching, nAMD patients received an average of 28 intravitreal injections (range 0–96), while DME patients received an average of 23 injections (range 0–63), with mean treatment durations of 45.38 (SD ± 38,80) weeks for both nAMD and DME patients. The most frequently used medication prior to switching was aflibercept 2 mg (52% in nAMD patients and 60% in DME patients), with an average of 13.12 injections per nAMD patient and 16.67 injections per DME patient. In switch patients, the last injection was received, on average, 4.8 weeks (range 4–20) prior for nAMD and 7.33 weeks (range 4–20) prior for DME. Before switching to faricimab, 24 nAMD-switchers received anti-VEGF therapy at four-week intervals. One patient was treated with Brolucizumab every 8 weeks. 16 DME-switcher were also treated every 4 weeks before the switch. 3 patients received the injection every 6 weeks (Brolucizumab and Triamcinolon) and one patient every 12 weeks (Dexamethason). No adverse events, particularly intraocular inflammation, were reported in the medical records after faricimab injections.

### Fluid volume and CST reduction

**Fig. 2:**
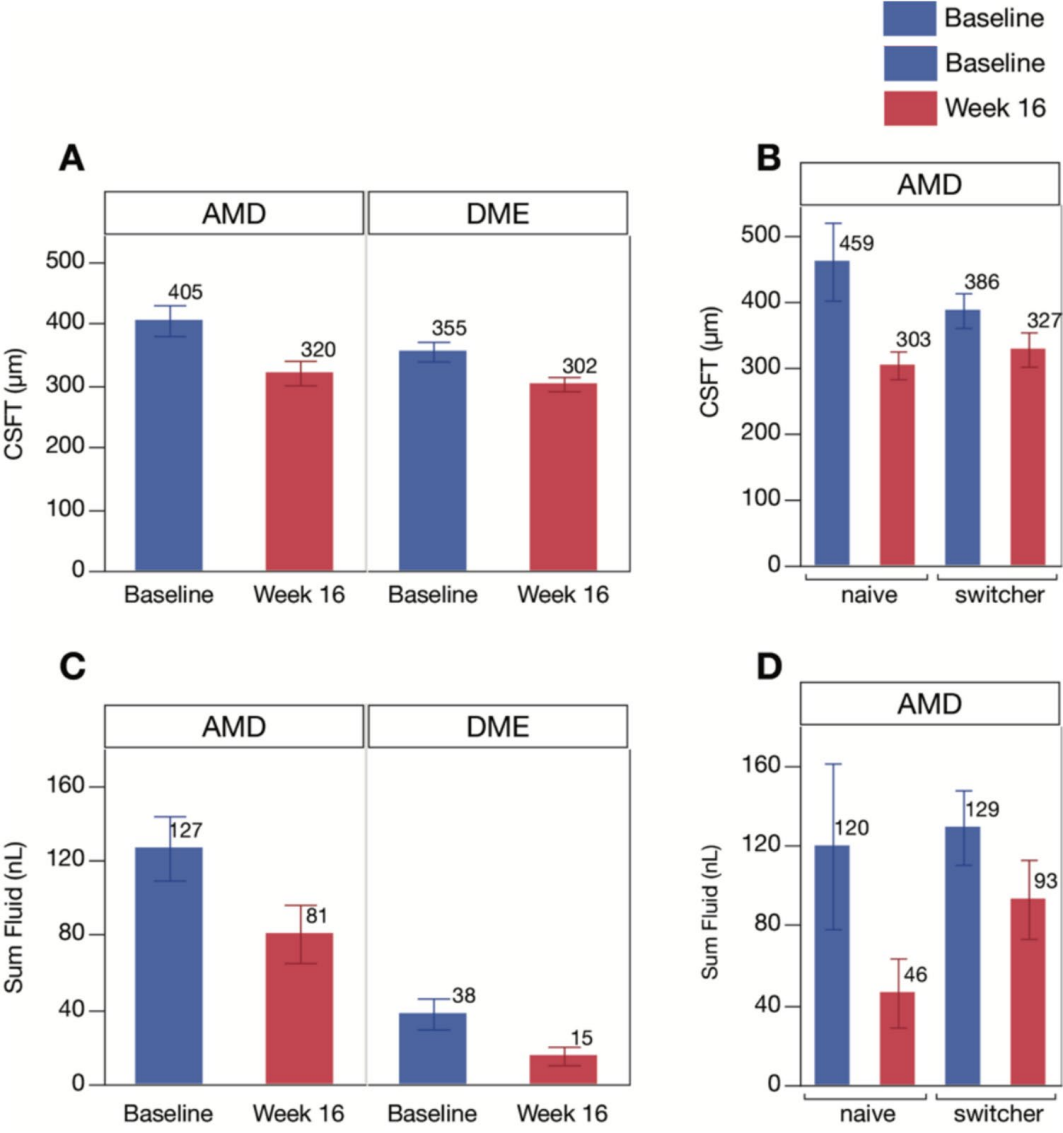
Average central foveal thickness (CST) and sum fluid (SF) volume reduction at baseline vs 16 th week in nAMD vs DME and in naïve vs switchers nAMD patients. **A** CST in nAMD vs DME; **B** CST in naïve vs switcher nAMD patients; **C** SF in nAMD vs DME; **D** SF in naïve vs switcher nAMD patients

Significant reductions in fluid volumes and CST at 16 weeks post-treatment were observed compared to baseline in both nAMD and DME patients. For nAMD patients, the mean baseline CST (µm) of 405.12 ± 24.95 reduced to 320.33 ± 19.80 (*p* = 0.0001). Similarly, in DME patients mean baseline CST of 354.97 ± 15.89 reduced to 302.41 ± 11.55 (*p* < 0.0001) (Fig. 2A). Mean SF (nL) also decreased significantly from baseline in both groups (126.68 ± 17.24 to 80.78 ± 15.56 in nAMD, *p* < 0.0001; 37.84 ± 8.31 to 15.28 ± 4.94 in DME, *p* < 0.0001) (Fig. 2C). When analyzing absence of SF in nAMD defined as a 2 nl cut off, then there were no patients in the cohort, whom reached the cut off at baseline. After 16 weeks it was 8.8% with absence of SF. In DME there were 4.8% of patients with less than 2 nl SF at baseline, at 16 h week there were 28.6%.

When comparing naïve and switch patients in nAMD group, CST (µm) decreased from 459.47 ± 58.89 and 386.55 ± 26.22 at baseline to 302.65 ± 20.92 and 326.69 ± 25.96 at 16 weeks, respectively. Similarly, SF (nL) reduced from 119.80 ± 41.51 and 129.16 ± 18.66 at baseline to 46.30 ± 17.32 and 93.19 ± 19.82 at 16 weeks, respectively (Fig. 2B and 2D).

### Compartmental fluid change

**Fig. 3:**
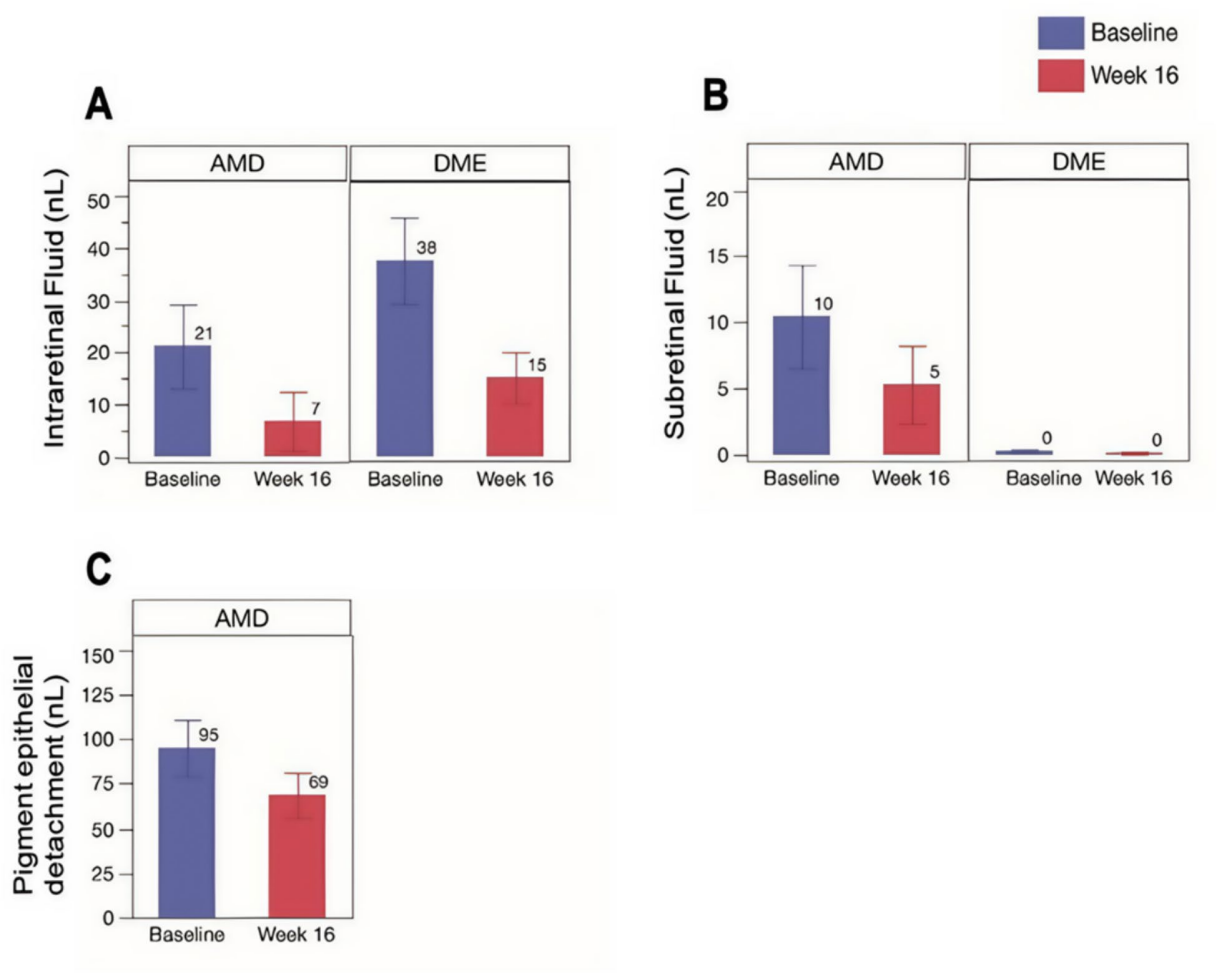
Fluid compartment change (SRF, IRF, and PED) between baseline and 16 th week. **A** IRF change in nAMD vs DME; **B** SRF change in nAMD vs DME; **C** PED change in nAMD patients

We observed significant reductions in both IRF and SRF among patients with nAMD and DME as shown in Fig. 3. Specifically, the mean fluid volumes for IRF (nL) decreased significantly from 21.17 ± 7.97 at baseline to 6.78 ± 5.66 at 16 weeks in nAMD (*p* = 0.0032) and from 37.54 ± 8.28 to 15.12 ± 4.95 in the DME groups (*P* < 0.0001), respectively. Similarly, mean SRF (nL) decreased from 10.46 ± 3.93 to 5.32 ± 2.93 at 16 weeks in nAMD (*p* = 0.0114) and from 0.30 ± 0.12 to 0.16 ± in DME (*p* = 0.0715), respectively. Additionally, mean PED volume decreased significantly from 95.05 ± 15.86 nL to 68.67 ± 12.53 nL for nAMD group (*p* = 0.0108). Analysis of compartmental fluid absence in nAMD and DME (2 nl cut off) shows an increase in patients with less than 2 nl of IRF, rising from 64.7% and 4.8% at baseline to 88.2% and 28.6%. SRF absence increases from 61.8% to 76.5%, respectively and PED absence from 5.9% to 11.8%, in nAMD. SRF absence (< 2 nl) was detected in all DME patients in the cohort either at baseline or after 16 weeks. Table 1 summarizes the compartmental fluid changes in treatment-naïve and switch patients in the nAMD group.

**Table 1:** Mean compartmental fluid change between naïve and switch patients in AMD group at baseline and 16-weeks

	Baseline	16-weeks
IRF		
naïve	13.95 ± 6.74	1.89 ± 1.64
switchers	23.77 ± 10.59	8.54 ± 7.69
PED		
naïve	104.62 ± 43.61	42.94 ± 16.36
switchers	91.60 ± 15.51	77.94 ± 15.75
SRF		
naïve	1.23 ± 0.66	1.48 ± 1.24
switchers	13.9 ± 5.20	6.71 ± 3.95

### Changes in SF vs CST

**Fig. 4:**
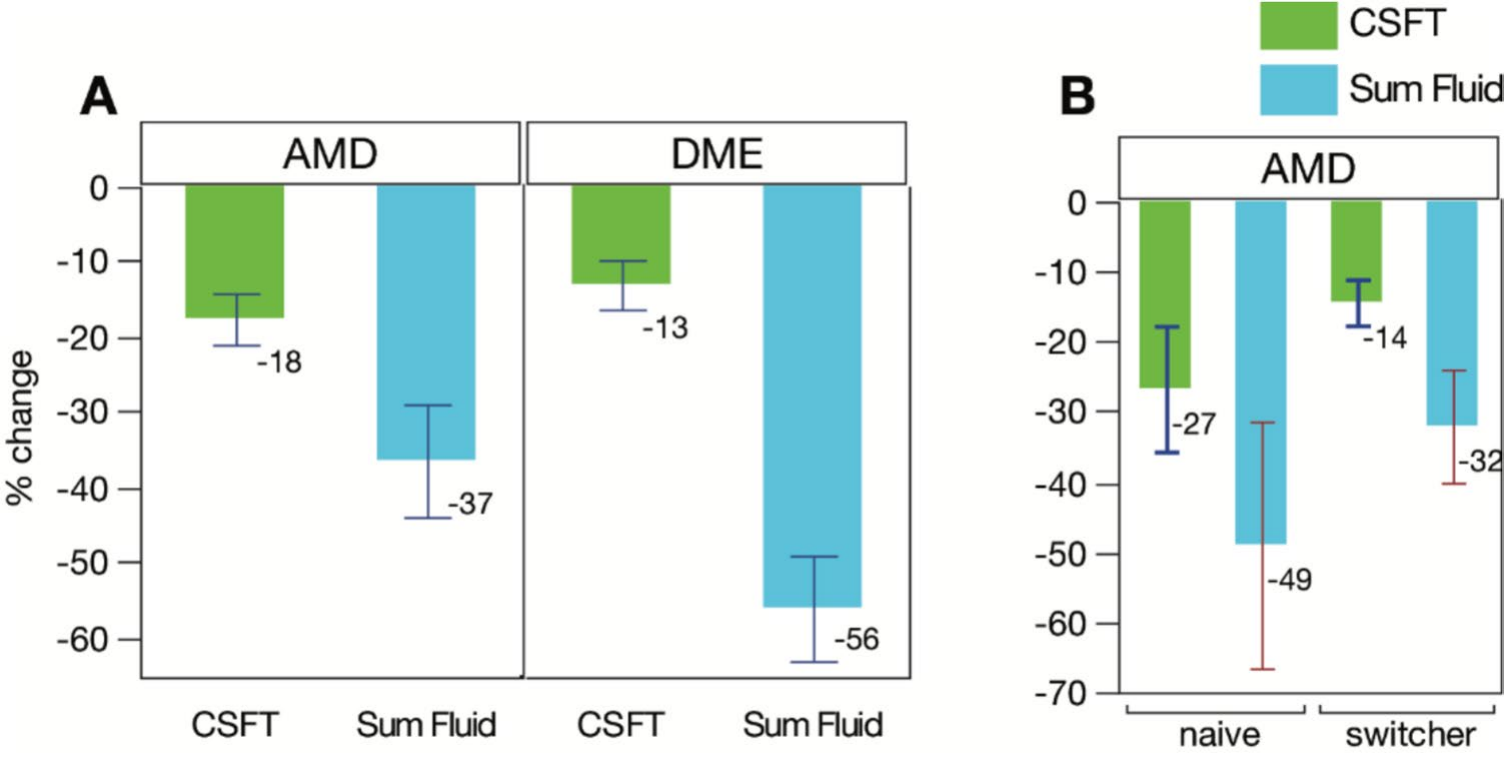
Comparison between percentage reduction of CST versus SF. **A** Percentage reduction in nAMD and DME patients; **B** Percentage reduction in nAMD naïve vs switch

Over a 16-week period, distinct differences were noted between SF and CST in patients diagnosed with nAMD and DME. Specifically, mean intraindividual percentage changes in SF were greater than those observed in CST (Fig. 4A). For nAMD, the mean intraindividual percentage change from baseline to 16 weeks was 36.5% for SF and 17.6% for CST. In DME patients, an even more pronounced difference was noted: SF change was 56.2% while CST change was only 13.1%, thus a roughly fourfold difference.

When comparing naïve and switch patients in nAMD, the naïve group exhibited a 49% reduction in SF and a 27% reduction in CST, while the switch group showed a 32% reduction in SF and a 14% reduction in CST (Fig. 4B). Thus, taken together for AMD, a roughly twofold difference between these 2 parameters remained constant withing these subpopulations.

### Correlation between CST and SF

**Fig. 5:**
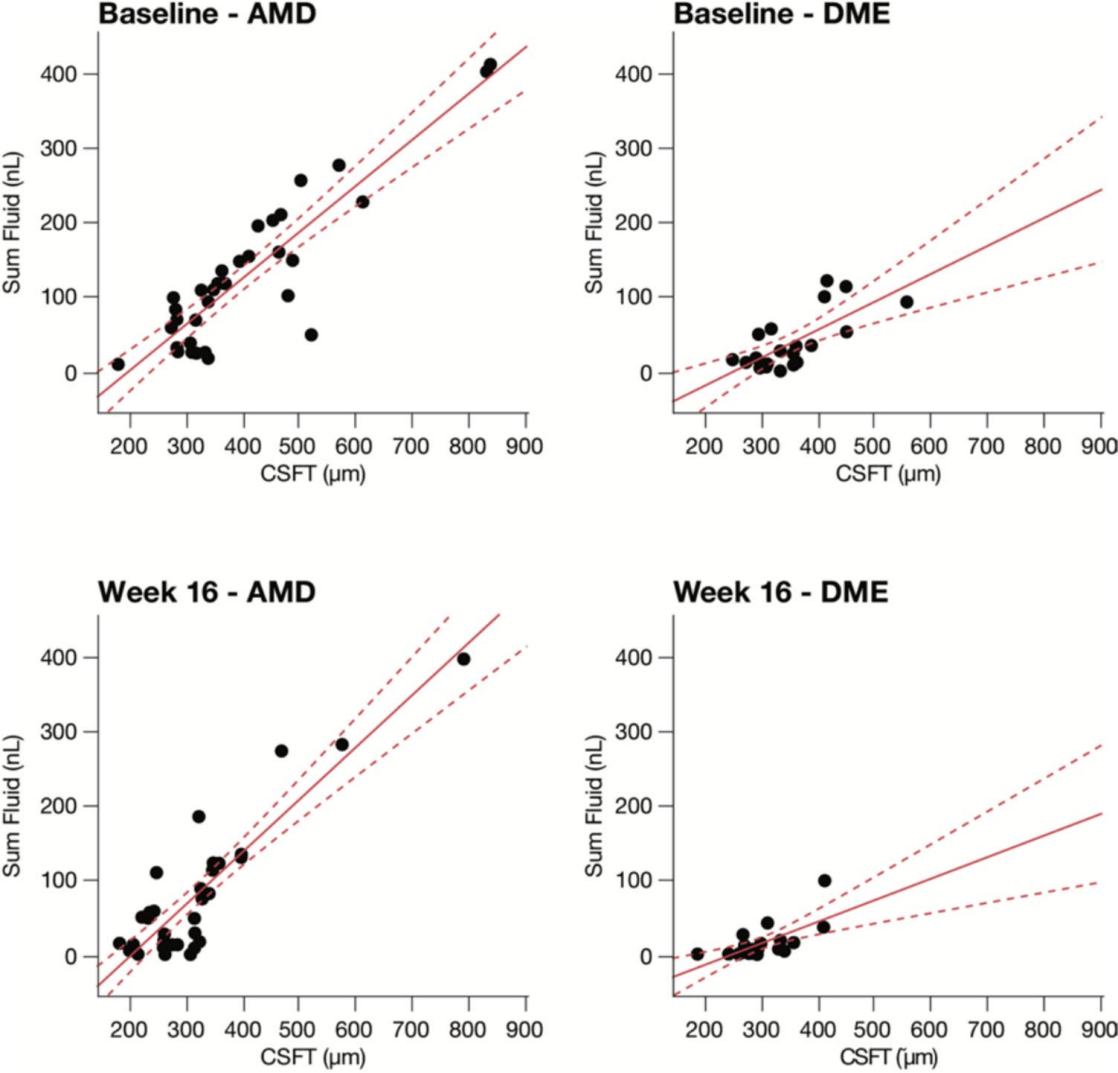
Correlation between CST and SF at baseline and 16-week

At baseline, the linear regression fit between SF and CST presented coefficient of determination (R-squared), was 0.799 (*p* < 0.0001) for nAMD and 0.511 (*p* = 0.0056) for DME, indicating a significant and positive linear relationship. By the 16-week follow-up, this relation showed similar pattern but with slight reduction of R-squared to 0.792 (*p* < 0.0001) for nAMD and 0.453 (*p* = 0.0044) for DME (Fig. 5).

### Corrected distance visual acuity (CDVA) change

For nAMD group, CDVA was 0.52 ± 0.07 logMAR at baseline and 0.46 ± 0.06 logMAR at week 16, showing an intraindividual change of − 0.06 ± 0.04 logMAR (*P* = 0.1452). Similarly, in DME group, CDVA was 0.48 ± 0.08 logMAR at baseline and 0.44 ± 0.07 logMAR at week 16, showing an intraindividual change of − 0.04 ± 0.05 logMAR (*P* = 0.2694).

Overall, improvements in visual acuity were not statistically significant, however 24 (71%) and 15 eyes (71%) showed visual acuity improvement or no change for groups nAMD and DME respectively. Accordingly, when nAMD switchers and naïve cases were analyzed separately, a small improvement in visual acuity with a slight statistical trend (*P* < 0.1) was observed in naïve eyes. The small sample size of naïve patients (*n* = 10) may limit the statistical power. These eyes showed a baseline visual acuity of 0.72 ± 0.23 logMAR, improving to 0.47 ± 0.14 logMAR at week 16 (*P* = 0.0625, Wilcoxon test), with an average intraindividual improvement of 0.25 logMAR. In contrast, switcher AMD eyes maintained stable visual acuity after treatment: 0.46 ± 0.06 logMAR at baseline and 0.45 ± 0.07 logMAR at week 16 (*P* = 0.4431).

## Discussion

The efficacy of retinal fluid resolution through intravitreal vascular endothelial growth factor (VEGF) inhibition is a benchmark for intravitreal agents. This study leveraged artificial intelligence-based quantification of retinal fluid to examine the efficacy of faricimab in treating nAMD and DME within a real-world context.

Using OCT and fluid measurement alone, without dilated fundus examination (DFE) and without visual acuity assessment, has been suggested to be a rapid, safe, and cost-effective approach to address the urgent requirement for timely treatment in busy OCT-guided injection centres [17]. However, physicians'interpretations and manual quantifications of fluid status are subjective, and thus, may be susceptible to diagnostic discrepancies [18]. Therefore, automated identification and standardized quantification of macular fluid is objective and thus capable of reducing interobserver variability by clinicians [19].

Our findings support integrating AI into clinical practice, showing that AI fluid quantification improves clinicians'ability to monitor disease and adjust treatment plans promptly. A post hoc analysis of DRCR protocol T using the Vienna Fluid Monitor revealed disparities in IRF reduction across anti-VEGF medications [20]. Thus, AI-based macular fluid quantification provides precise, reproducible data that improve diagnostic accuracy and treatment efficacy.

Faricimab, a novel bispecific antibody targeting both VEGF-A and Ang- 2, aims to manage retinal diseases like nAMD and DME by simultaneously inhibiting angiogenic and inflammatory pathways, thus contributing to vascular stability [21]. In this study, there was a notable anatomical retinal improvement after the faricimab injections in patients with nAMD and DME, as indicated by reductions in CST as well as IRF, SRF and PED observed at the 16-week follow-up. This is in line with findings from key clinical trials for nAMD and DME, demonstrating faricimab’s improved efficacy in reducing CST, compared to existing anti-VEGF therapies [6].

While clinical trials often have strict criteria limiting generalizability, our cohort's anatomical and functional outcomes with faricimab align with real-world studies showing reductions in CST and fluid [22–29]. In contrast to the aforementioned studies, our work presents quantitative data on the impact of faricimab on retinal fluid dynamics. Specifically, we observed a 36.5% and 56.2% reduction in fluid accumulation in nAMD and DME, respectively. The reduction in SF was approximately 2 and 4 times greater than the reduction in CST for nAMD and DME, respectively. Existing research often examines the switch from aflibercept to other therapies, our study includes patients who switched from a variety of anti-VEGF drugs. While the former is more conceivable from a statistical standpoint, our broader approach offers more robust and generalizable data, enhancing the relevance and applicability of our findings to a real-world scenario.

Using the RetInSight Fluid Monitor®, we analyzed overall fluid volumes and their distribution in disease compartments (IRF, SRF, and PED) in the central 1-mm retinal zone. Our analysis revealed significant reductions in both IRF and SRF among patients with nAMD and DME. Moreover, PED decreased significantly by 27.8% in nAMD group, although the reduction observed was less pronounced compared to the reductions in IRF and SRF. However, the consistency of a PED varies as it can be predominantly serous, fibrovascular, drusenoid or a combination thereof, thus reflecting the variability of VEGF inhibition. This is consistent with an AI based post hoc quantitative analysis of macular fluid volumes performed on 1095 nAMD patients from the HARBOR trial, which showed distinct response patterns for each fluid compartment following ranibizumab therapy: IRF exhibited the most significant and rapid resolution, followed by SRF, with PED resolving to a lesser extent and at a slower pace [30].

We also correlated the CST with SF. As we calculated correlations between 2 measurements that are directly related to the retinal volume at the macula area (CST and SF), we indeed expected the observed relationship. However, what is interesting in this result is that approximately 20% (nAMD) and 50% (DME) of the SF variability cannot be explained by CST variability at baseline and at 16 weeks post-treatment. When we compare both at baseline and at 16-weeks: the coefficient of determination (R^2^) from the linear regression was weaker in DME group, either at baseline or at 16 th week follow up, with (R^2^ = 0.511, 0.453), respectively. In contrast, Pawloff et al. observed that patients with nAMD exhibited a weak-moderate correlation between CST and fluid volume measurements in the central 1 mm (0.107–0.569) and patients with DME exhibited a moderate-strong correlation between IRF and CST (0.668–0.797) [14]. However, their definition of CST relied on ILM to RPE surface, whereby our study utilized ILM to BM. Assessment of OCT-based central subfield thickness (CST) as a quantitative measurement has been extensively employed as a proxy for disease activity, alongside traditional best-corrected visual acuity (BCVA) testing in large randomized controlled trials (RCTs) [31, 32]. The observed lack of perfect correlation between CST and SF highlights the complexity of assessing treatment efficacy solely based on retinal thickness measurements in nAMD and DME, and we endorse that monitoring retinal fluid volume may offer more granular insights in this field.

Comparing treatment-naïve nAMD patients with switch patients, a review by Nasimi et al. analysed 22 real-life studies [33]. They describe evidence in visual acuity improvement and CST reduction in naïve patients and a stable visual acuity despite anatomical improvement with longer treatment duration in switch patients. Our data align with these findings, showing a trend toward visual acuity improvement in naïve eyes, while switch patients maintained stable visual acuity. The borderline p-value in visual improvement among naïve eyes could be due to the relatively small sample size, which limits the ability to reach statistical significance. Anatomically, the naïve AMD patients show a 50% reduction of SF after 4 injections of faricimab, in contrast to 33% in switch patients.

In this real-world analysis, the improvements in visual acuity were not statistically significant– despite the significant anatomical changes–this is consistent with other recent evidence in the literature [34–36]. The limited improvement in CDVA can be attributed to the chronic nature of the disease and the resultant structural changes in the retina. Since a majority of our patients were switchers (74% eyes with nAMD and 95% eyes with DME), with an average of 28 and 23 prior intravitreal injections for nAMD and DME, respectively, it is likely that they could already have suffered photoreceptor damage.

In our study, the 2-nanoliter threshold for the absence of fluid was selected based on the lower sensitivity range of the RetInSight Fluid Monitor algorithm. However, it is important to note that this fluid volume represents a clinically negligible amount. Other AI-algorithms offer varying spectrums of sensitivity in fluid detection. No consensus exists currently in the field on what would be a clinically meaningful threshold level as a proxy for disease activity in real life.

This study has limitations, including the retrospective single-center study design and the relatively small number of participants, which limit the applicability of the findings and reduces the statistical power for conducting multiple analyses. Moreover, we only present short-term data on the effect of faricimab load on retinal fluid, so we cannot make any inference toward treat-and-extend regime. Maturation bias is another potential limitation for the switch group in this cohort due to the patients'history of prior treatments. All patients received the prior treatment strictly with comparable intervals. Only the intervals before switching to faricimab varied (nAMD SD ± 3,6 weeks; DME SD ± 4,21 weeks). However, since the percentage fluid reduction and not only the CST was calculated, this point is negligible. We did not exclude patients with presence of fibrosis and/or outer retinal atrophy in our analysis. This can explain the limited functional improvement despite significant anatomical improvement. Another limitation is the AI-based segmentation. Algorithms sometimes struggle to accurately delineate the inner limiting membrane (ILM) in cases with significant cystoid edema. Moreover, CST measurements may encompass different distances, such as from the ILM to the external boundary of the neurosensory retina (NSR) or to the retinal pigment epithelium (RPE), depending on the presence of subretinal fluid (SRF) and subretinal hyperreflective material (SHRM), or fibrovascular pigment epithelial detachment (PED), each representing distinct pathomorphological entities [37–39]. The in-machine segmentations of Spectralis and Cirrus show in general poor performance in older version when fluid or drusen are present, while the Retinsight Fluid monitor layer segmentation excels in fluid cases even in extreme situations with bad scan quality or large deformations. In the current small data set, all CST values (and their corresponding segmentation) have been checked on a b-scan level to confirm that they are accurate. Difficulty in identifying the foveal center in macular edema can also lead to inaccurate CST values by the Spectralis device, despite robust segmentation by the FM algorithm itself [14, 18].

## Conclusion

In this real-world analysis, faricimab was found to significantly reduce retinal fluids and maintain vision in nAMD and DME patients. The reductions in fluid volume were more pronounced than changes in CST, indicating that AI based fluid volumetry may provide a more sensitive measure of treatment efficacy than traditional metrics like CST. Further validation is recommended through research with larger, real-world cohorts.
